# From harassment to disappearance: Young women’s feelings of insecurity in public spaces

**DOI:** 10.1371/journal.pone.0272933

**Published:** 2022-09-07

**Authors:** Ariagor Manuel Almanza Avendaño, Martha Romero-Mendoza, Anel Hortensia Gómez San Luis

**Affiliations:** 1 Faculty of Human Sciences, Autonomous University of Baja California, Mexicali, Baja California, Mexico; 2 Directorate of Epidemiological and Psychosocial Research, National Institute of Psychiatry Ramón de la Fuente Muñiz, Mexico City, Mexico; San Diego State University, UNITED STATES

## Abstract

**Background:**

There are processes of gender socialization that increase the vulnerability of young women against a continuum of threats in the public space. This study explored the feeling of insecurity in public spaces among young women in a city located near the northern border of Mexico.

**Methods and findings:**

This study was based on the tradition of grounded theory. Purposive sampling was used, and 24 group interviews were conducted with junior high school, high school and university students to understand the conditions that favour the emergence of the feeling of insecurity, its psychosocial consequences and management strategies. A computer-assisted qualitative analysis was performed using MAXQDA 18 software. The study showed that street harassment was manifested mainly as sexual harassment but generated a continuous threat of feminicide and disappearance. Young women experienced discomfort and restrictions on mobility, and they had to assume individual responsibility for their safety in the absence of support from the state.

**Conclusions:**

The interaction between gender and age determines the vulnerability to crime in urban spaces. Future studies could analyse the chronic impact of this continuum of threats and develop psychosocial interventions that promote the empowerment of young women.

## Introduction

Crime generates a sense of threat, vulnerability and daily concerns about the risk of becoming a victim [1]. Fear of crime is an “emotional response of dread or anxiety to crime or symbols that a person associates with crime” [2]. People may experience fear based on the crime rate in their locality and the degree of direct and indirect victimization they experience [3].

Several studies have shown that women express greater fear of crime than males [1, 4, 5], although exposure to crime in public spaces is usually lower [6]. One possible explanation for this paradox is that fear is related to the perceived vulnerability to a physical attack due to characteristics such as sex, size or age. Vulnerability also has a social component, since there is greater potential exposure to crime according to attributes such as ethnic group, socioeconomic status, educational level or marital status [7]. Vulnerability, in turn, is situational, since there are places and moments of greater risk to which they may be more exposed, depending on daily activities, lifestyle and mobility in the city [8]. Based on these vulnerabilities, women are constantly evaluating the probability of being exposed to crime, their ability to control risky situations and the severity of the anticipated consequences in case of becoming a victim [9].

Another explanation for the higher levels of fear is that in the family and community spheres, women are socialized to fear crime. They receive messages about their physical vulnerability, the threat of sexual violence and the risks of victimization in public spaces. The expression of fear and concern for their own safety is constructed as an appropriate gender response. They are blamed if they do not adopt protection and risk avoidance measures, such as restricting their mobility, changing their lifestyle or seeking accompaniment [10]. The fear of crime simultaneously represents a fear of aggressions perpetrated by men, particularly in public spaces. This fear subordinates women and keeps them in an invisible prison, since their freedoms are limited to reducing the probability of being victimized [8].

Women are more exposed to sexual crimes than males. The threat of sexual crime is a way of maintaining a woman’s subordinate status and controlling their behaviour in public spaces; it is a spatial manifestation of gender-based power relations [11]. The shadow of sexual assault hypothesis proposes that in women, the fear of sexual crimes such as rape increases the overall fear of crime [12]. Fear of rape is an emotional and behavioural response to the possibility of rape victimization. It involves behavioural adaptations to minimize risk, such as restricting mobility, which affects participation in the workplace and educational or recreational settings [13]. Rape victims are mainly young, sexually mature, attractive women [14]. Fear is experienced by both direct victims and those who have had a vicarious experience [15]. Fear of this type of crime can be worse at night due to less availability of help, less bystander deterrence, and inability to see potential offenders or risks [16].

Rape is a contemporary perceptual offense, as it is a serious crime that evokes fear due to cognitive connections with other crimes. It is conceived as a “master offense” for women because it has the greatest connection to other, potentially violent, crimes [17]. These violent crimes involve physical aggression, both sexual and non-sexual. Fear of sexual assault, including rape, is associated with fear of nonsexual violent crimes that involve face-to-face contact, such as homicide or assault [6, 18].

Contemporary perceptual offenses can vary depending on the crimes that occur in the local context. In Mexico, feminicide is another crime that causes fear in women. A murder is typified as feminicide when it occurs in the family context or in an intimate partner relationship; there is sexual violence or mutilation before the murder; or when the body has been exhibited or discarded in a public space [19]. Although globally the term femicide is used more frequently, the concept of feminicide has been developed to indicate that murders of women are not circumstantial or random acts committed by violent subjects but are part of a process and have a systemic nature. Feminicide is an expression of patriarchy, which establishes asymmetric relationships based on gender that are reproduced in multiple fields [20].

The increase in feminicides and forced disappearances in places on the northern border have been linked to the activity of war machines related to organized crime [21]. War machines are diffuse and mobile organizations of armed groups that merge or divide according to their tasks and circumstances [22]. They maintain different relations with the state, from autonomy to cooptation and incorporation. These relations arise especially in contexts where the state has lost the ability to maintain public order. Women can be victims of these groups due to their involvement in high-risk activities, such as the transportation, distribution or sale of drugs; conflicts between criminal organizations; and involvement in ‘shadow economies’ such as human trafficking, prostitution and other aspects of the sex industry [23]. However, violence against women is also exercised daily by men who do not belong to criminal groups. Fear of crime is amplified when women are also exposed to symbolic or communicative aggressions and minor crimes.

Fear of crime among women is related not only to master offenses but also to the fear of more common expressions of sexual objectification, such as sexual harassment in the street by unknown men [8]. Sexual harassment consists of unwelcome sexual advances, requests for sexual favours or other intrusive behaviour of a physical or verbal nature with sexual overtones [24]. When it is restricted to sexual harassment in the street, it implies experiencing unwanted sexual attention by strangers in public spaces, including public transportation [25]. The unwanted nature of the encounter implies a sexualization of the person being harassed and discomfort [24].

Sexual harassment in the street encompasses a wide variety of behaviours that can occur face to face or from a vehicle, individually or in groups, and there are several types: verbal (e.g., “catcalling”, kissing noises, whistles, asking for a smile, unwanted conversation requesting a woman’s name, a date or telephone number, which may persist after having been rejected; hostile comments about gender or sexist speech, such as sexual names or graphic sexual comments about appearance; sexual demands or threats); nonverbal (staring, leering, sexual gestures, indecent exposure, public masturbation); proximity (stalking or following someone, honking the horn); or that involve physical contact (being too close, rubbing) [24–26]. Some classifications include types of sexual assault such as unwanted sexual touching, sexual abuse and rape [11]. For the purposes of this study, these types are not considered as harassment but rather as part of a continuum of sexual aggressions that can be suffered in the street.

Difficulties have been noted in the classification of conduct as sexual harassment. For example, harassment can be subtly manifested as greetings and compliments, since the rules of civility can be used to hide harassment. The ambiguity of behaviours that are not overtly sexual, vulgar or hostile requires considering the context for their interpretation [27]. Subjectivity also influences the interpretation of forms of intrusion, such as sexual harassment, as well as myths or stereotypes [11]. Self-recognition of sexual harassment based on personal experience is associated with overrecognition, while using legal definitions is associated with underrecognition. In addition, the victim may have been sexually harassed based on these definitions but not recognized as a victim [28].

Therefore, criteria have been proposed for the recognition of sexual harassment, including the unwanted nature of the act, the sexual intention of the harasser, the existence of a pattern or repetition, behaviour that is intrusive and of a sexual nature, and the generation of threat or fear [28]. In the case of subtle forms of sexual harassment such as greetings or comments, additional criteria can be used to address pragmatic aspects of communication instead of focusing exclusively on the content, for example, previous personal experience with sexual harassment, intrusive behaviour that causes discomfort and demands attention and actions of strangers that make one feel like an object or prey [27].

Starting in adolescence, women begin to perceive themselves as vulnerable to sexual harassment in public spaces, and their main fear is sexual violence [29]. A study conducted in Mexico of women aged 12 to 17 years showed that more than 65% had experienced harassment, starting at an average age of 10.8 years. The types of harassment with the greatest impact were sexual exposure and persecution, which were associated with a fear of being kidnapped or raped [30].

Women can also experience incivilities in the street by strangers, considered low intensity behaviours, such as walking too closely, invading someone’s personal space, pushing in line, shouting and rudeness. While there are incivilities linked to gender such as insults based on stereotypes or exposure to sexist material, it is possible that incivilities not linked to gender are also interpreted as sexual harassment. In particular, women who have experienced previous sexual harassment or diverse forms of sexual violence may perceive these incivilities as threatening, experience fear and thus avoid public places where there is a presence of male strangers [26].

Both sexual harassment and incivilities represent a breakdown of an implicit interactional order, violating the norm of not interacting with strangers in public spaces if they do not know each other. Groups of low social status, such as young women, are perceived as approachable people in the street [11]. Expressions of affection are usually intended for people who know each other well, but this norm is violated when a stranger expresses affection without being in a previous relationship with the person. There is an attempt to define the relationship with women based on a fantasy of interactional involvement and heterosexual intimacy. The expressions of affection are not reciprocal because the position of power is used to force interaction with the person [27]. The intrusion may be sexually motivated, but the difference in power is used to approach the person on the street [31].

Interactions in public spaces are influenced by gender regulations. The streets become a territory of male dominance where men prove their masculinity, approach women considered to be of lower status or out of role, and women modify their behavior to reduce the risk of sexual harassment [32]. Street remarks are often initiated by males. Although these messages can be ambiguous, they are perceived as offensive and intrusive when there is not enough involvement with the sender, they have inappropriate content or length, and they imply evaluation or objectification. For women, receiving street remarks requires an exercise in self-control. Gender regulations force them into a position where they simultaneously have to respond politely and ignore comments to appear inaccessible to the general public. For men, making street remarks also represents a process of socialization towards rejection [33].

### Fear of crime: Contextual conditions and consequences

Fear of crime is not solely constructed from the interpretation of the risk of becoming a victim of any of the offenses (including incivilities, violent and non-violent crimes) that occur in public spaces. Fear also manifests as a set of conflicts and concerns about the neighbourhood, the threat of social problems and the position of the subject in her social world. It expresses a public concern about local conditions that contribute to vulnerability [34], such as the level of social inequality, trust in the police and the legal order [35, 36] and signs of disorder in the community [1, 37].

Community disorder is an important contextual condition associated with fear of crime. Among the indicators of deterioration of order are graffiti, the consumption of substances in public spaces, abandoned buildings [17], the presence of garbage and homeless people [38]. Other indicators include the presence of illicit markets (prostitution or drug trafficking), social disorder (begging, public intoxication, or the presence of gang members) or physical disorder (for example, poor lighting or buildings in ruins). Community disorder, which is common and visible, sends the message that social controls are weak. Criminals perceive that there is no community organization to respond proactively, which feeds criminal activity, which is usually less common or visible. People pull back from community life, which contributes to both the increase in crime and community decline [39]. Fear of crime is reduced when there is familiarity with the neighbourhood and members of the community [8], as well as social integration [40], trust, building of networks and social capital, collective efficacy and maintenance of community norms [17]. Physical incivilities are one of the main community-level predictors of fear of crime, along with collective efficacy [5].

Fear of crime has various psychosocial consequences. In terms of health, it can influence the appearance of diseases directly or indirectly. The direct route consists of raising the level of stress, which in turn causes physical changes such as accelerated heart rate, which may increase the risk of developing cardiovascular diseases. The indirect pathway occurs when people resort to unhealthy behaviours to manage stress, such as smoking, which increases the risk of disease development [41]. It also contributes to the deterioration of mental health, as it is related to anxiety, somatization, depression and psychological distress [42]. Finally, it affects mobility outside the home, restricts recreation, socialization or health care activities, causing social isolation and decreased life satisfaction [43, 44]. At the community level, trust and cohesion deteriorate [45].

### Feeling of insecurity

Despite its usefulness and relevance in the criminological field, there have been multiple criticisms of the concept of fear of crime. The primary criticism is that it assumes there is a primary way of reacting to crime instead of considering a broader spectrum of responses. For example, people can experience varying degrees of concern about crime, from the absence of concern to feeling anxious concern [46, 47]. Likewise, focusing exclusively on fear ignores the possibility of a wide range of emotional responses to crime, such as anger or sadness [48]. Another criticism is that fear of crime is considered mainly dysfunctional based on the degree to which it affects quality of life. However, fear can be useful and adaptive when it allows one to prepare for threats and adopt routine precautions without affecting the quality of life and even improving security [46].

The concept of fear of crime privileges the notion of an individual subject who responds cognitively, emotionally and behaviourally to the threat of victimization. However, subjects interpret and respond to crime through symbols that they share with their reference groups; that is, they participate in a collective construction derived from social processes associated with the interpretation of risks [49, 50]. In addition to the individual concern about becoming a victim, the subjects simultaneously manifest a public concern about various aspects of the sociocultural context that frame their fear of crime [34, 45, 47].

A concept associated with fear of crime is the perception of insecurity. There is an objective dimension of insecurity, based on collective information on criminal incidence and urban disorder. At the same time, there is a subjective dimension of insecurity, which encompasses fear of crime, perceived risk and security behaviors. The perception of insecurity is an individual assessment of the environment in which the person is inserted, based on the interpretation of risks, dangers or threats, in specific times and places [51]. It is based on subjective reactions to the risk of crimes, which may not coincide with crime incidence figures, but feed on the uncertainty of the social context [52].

The perception of insecurity has been considered as a concept that integrates the cognitive response of evaluation of the probability of experiencing a threat or being victimized, the emotional reaction to such a threat, as well as protective behaviors. It has been argued that the perception of insecurity is not only based on specific or situational threats to the individual, but also on the general perception of the social environment [53].

The concept of perceived insecurity can be ambiguous, fluid, difficult to operationalize, affected by a great individual variation in the interpretation of the environment and paradoxes arise when objective figures do not coincide with subjective perception. For example, there may be a greater perception of insecurity in places with a low crime rate. The night is feared more, even though more crimes are committed at other times of the day. There is greater fear of bodily offenses but there is a higher proportion of crimes against property. People who have experienced previous victimizations may report lower levels of fear than those without experience of victimization. Furthermore, people perceived as threatening may be more vulnerable to victimization [54].

The perception of insecurity has a spatial dimension, given that the perception of physical and social disorganization influences its construction [55]. It has been noted that the spaces most vulnerable to crime arise from the fragmentation of the urban fabric, which separates the city into used spaces and peripheral spaces. The conditions of poverty, informality, self-construction, subsistence culture, real estate devaluation, institutional abandonment, or precarious relations with the State, contribute to the control of territory by criminal groups [56]. There is also a media dimension, since the influence of the overrepresentation of crimes with a high emotional impact on the perception of insecurity is recognized, despite the low probability of occurrence of these crimes [54].

Other environmental elements that contribute to the construction of insecurity perception are the mechanisms of informal social control that arise from the strengthening of social ties. These types of links allow the generation of trust between the members of the community, the capacity for association, collective participation in the surveillance and recovery of public spaces, as well as in the collaboration with the institutions for the solution of security problems. In a complementary way, the notion of insecurity implies the construction of social actors as threatening or dangerous figures, urban alterities that are valued as socially despicable [55].

In this study, the concept of feeling of insecurity will be used, which does not solely refer to the emotional response to crime; it is also based on the premise that emotion itself is not something that is accessed but rather refers to the way in which it is expressed through the discourse on crime. This discourse addresses the current dangers in the social environment and does not necessarily require that people have suffered direct victimization experiences. It considers that emotions are part of a representation of the social world and are not separated from reason. Emotions are contextual, fluctuating and of variable intensity [48].

The concept of the feeling of insecurity is considered a network of representations, discourses, emotions and actions [57]. People experience emotions about crime that are linked to an interpretive framework that allows them to give meaning to their experience. The feeling of insecurity also has a political implication given that the interpretive framework that people construct forces them to take a position on the causes of crime and the strategies required to control it and to demand that the state guarantee an acceptable level of protection [48]. In the present study, the feeling of insecurity is not built exclusively around crime but encompasses broader situations of aggressions or incivilities that are not necessarily classified as crime.

The interpretive framework on crime and the emotions linked to it are also linked to the generation of strategies for personal risk management in daily life. There are avoidance strategies, such as avoiding areas and restricting exits, in addition to defensive strategies, such as the use of technological devices and private security services [48]. The use of the strategies forms protective routes which are spatially and temporally delimited. Changes in movement occur so that certain areas of the city are not visited or the way in which these areas are navigated is altered. Because protective actions are directed at the body itself and involve a transformation of daily life, oscillations can occur. After violent events or critical periods, avoidance or defensive protection strategies increase, but they decrease when the situation in the local context stabilizes. Therefore, there is a periodic reappraisal of threats based on personal experience in the urban space and the comparison of present security with that of the past [57].

The purpose of this study is to understand the feeling of insecurity by a vulnerable group in the public space: young women from a city located on the northern border of Mexico. This group is considered vulnerable due to the intersection of gender and age [11], both of which are associated with a greater exposure to aggressions and crimes by men within the framework of asymmetric relationships [26]. Offenses committed in the public space and the contextual conditions that affect the development of the feeling of insecurity are explored. The elements that make up the feeling of insecurity are analysed, including meanings, emotions and strategies. Likewise, their psychosocial consequences are identified.

### Study site

The present study was conducted in the city of Mexicali, the political capital of Baja California, which is located on the border with California, United States. The city has more than one million inhabitants and has expanded in recent years due to population mobility associated with internal and international migration processes. Among the main economic activities are agriculture, livestock, commercial and service activities, as well as the maquiladora (manufacturing or textile assembly) industry. Binational cultural and economic exchange has also involved the exchange of drugs and weapons, human trafficking and sex tourism. Mexicali has been identified as a destination for adolescents in the sex industry [58].

The perception of insecurity in the city (58.1%) is lower than the perception in the state or in nearby cities such as Tijuana [59]. In 2019, the local crimes with the highest incidence in Mexicali were home robbery, robbery of passers-by on public roads, intrafamily violence and drug dealing. In recent years, the neighbourhoods with the highest crime rate have been concentrated in certain sectors of the city centre but mainly on the periphery of the city [60]. The reports of intentional homicides and feminicides have remained low and stable since 2015 [61]. In the case of feminicide, under-recording is likely since figures three times higher have been reported in the locality [62]. As of October 2017, 227 disappearances had been recorded, including 74 women in the age range of 0 to 29 years. Most of these cases were concentrated among adolescents aged 15 to 17 years [63].

## Methods

An interpretive study was carried out in the constructivist grounded theory tradition [64] to understand the symbolic process of constructing feelings of insecurity through the objective conditions of young women’s experience. The participants were men and women from public schools in the urban area, although this study only reports the results of young women. Another criterion for the selection of the participants is that they have attended schools located in neighbourhoods in various areas of the city with a crime incidence higher than the third quartile in the last five years [60]. The selection of public schools was due to issues of access and to the fact that in areas with high crime rates, public schools were predominant, and the students were more exposed to high-risk situations. Theoretical sampling was used to contrast the educational level (secondary, high school and university) and the region of the city (north, south, centre, east, west).

The review board that provided the financial support for the study (PRODEP 511–6/2019.-7930) was responsible for the approval of the research and of the study’s research methods. The authorities of each school were approached to present the project and request their authorization for the participation of the students. If the authorities agreed to participate in the study, the consent of the students’ guardians was then requested. If the parents of the young women gave their consent for their daughters to participate, afterwards, each young women also gave their written informed assent. Informed consent was requested directly from university students. Informed consent was reviewed with the participants; it established that their participation was voluntary and that they could choose not to participate or to withdraw from the study if they considered it necessary, even when their guardians had given consent in the case of minors. The participants’ permission to audio-record the interviews was requested. The names of the students were not requested to ensure anonymity. Before conducting the interviews, a questionnaire was individually administered to collect sociodemographic data, including socioeconomic indicators [65].

A total of 168 students with an average age of 16.7 years (SD = 3.23 years, range = 13 to 24 years) participated. Junior high school students had an average age of 14.36 years (SD = 0.746 years), high school students 16.34 years (SD = 1.23 years) and university students 21.77 years (SD = 3.11 years). More than half the participants (54.2%) lived in a nuclear family with both parents; 29.7% in a single-parent household; 7.7% in a blended family; and the remaining 8.4% in other family arrangements. The university students were studying for careers in communication, law, education, business administration and physical education. Forty percent of university students had a job. The distribution of the participants in terms of schooling and socioeconomic level is shown in Table 1.

**Table 1:** Sociodemographic characteristics of the participants (n = 168).

Characteristics	Percentage
Schooling	
Junior high school	41.1%
High school	38.1%
University	20.8%
Socioeconomic status	
High	17.3%
Medium	74.4%
Low	8.4%

Source: Prepared by the authors.

Twenty-four interviews were conducted in schools with groups of five to seven students, with a maximum duration of one hour. The interviews were conducted by the first (male) and third (female) author. Both were researchers at a public university at the time of the study and had training in Clinical Psychology and qualitative methods. There was no relationship with the participants prior to the study. When contacted, they were informed of the reasons for the study. Only the participants and the researchers were present during the interviews. One young woman refused to participate, because she preferred not to talk about the situation in her neighbourhood.

Ten group interviews were conducted with the junior high school students, ten with the high school students and four with the university students. A thematic guide was used to develop the interviews. The thematic axes were the concept of insecurity, the situations and actors associated with insecurity, the psychosocial consequences of insecurity and strategies for managing the insecurity. The interview guide used in this study can be consulted in the S1 Appendix.

A single interview was conducted with each group. The interviews were audio recorded. Field notes were made after the interviews. The termination of the interviews was decided by the saturation of the data. Grounded theory was used to understand the constructive process of the feeling of insecurity and to illuminate situations of people without public voice [66]. From constructivist grounded theory, the importance of the positionality and reflexivity of the researcher is considered since he or she participates in the joint construction of the data [67]. A review of the literature prior to the study can be performed in an open, critical, analytical and uncompromising way. Because the authors have carried out previous studies on feelings of insecurity related to organized crime with adult populations, it was not necessary to research the literature again to develop the theoretical framework [66].

Computer-assisted analysis was carried out by two data coders using MAXQDA 18 software. Initially, open coding was performed, line by line. As the codes were contrasted with the new data, the codes that emerged repeatedly were grouped into broader categories or subcategories. Subsequently, relationships between categories were identified, which were contrasted by theoretical sampling. Memos were used to conceptualize the way in which the categories were connected to account for the experience of young women in public spaces [68]. Finally, a core category was identified with which the rest of the categories were connected [66]. This process seeks to generate a substantive theory about a phenomenon in a particular situational context [69].

Various criteria were used to ensure the rigor of the analysis. In terms of credibility, the participants were allowed to guide the data collection process, exploring emerging issues through the interviews. The theoretical construction generated with the participants was also verified through direct questioning, and an attempt was made to use their terms for the creation of the categories. The personal perspectives of the researchers and insights into the study problem were identified through analytical files such as memos and personal diaries, in addition to monitoring the use of previous literature [69]. Triangulation of the results was performed by two of the authors.

## Results

### Contextual conditions of the feeling of insecurity

Feeling insecure in the city depended to some extent on exposure to crime and aggressions that were perceived as threatening. One crime the participants experienced directly was robbery (n = 10, 41%), including the robbery of personal belongings, a house or a car. These crimes were the most common according to government figures for criminal activity in the locality [60].

The most common situation that the participants experienced daily was sexual harassment (n = 23, 95.8%), both on the street and when using transportation, as has been previously reported [70]. They mentioned different behaviours of sexual harassment, including nonverbal harassment through leering or waiting for young women to pass by specific places to watch them. This type of harassment is commonly expressed verbally through “cat-calling” or offensive shouts, whistles or “throwing” kisses. The situation of being approached by strangers who begin to speak to them or try to converse in a more intimate way is also identified as sexual harassment. More serious manifestations were exhibitionism, masturbating in front of them or even touching them when walking on the street or in public transportation.

I used to like walking around here, but it started to scare me because you were alone and not even wearing provocative clothes, but you were wearing normal pants and a normal shirt and makeup, and the guys passed by in the car and yelled things at you or they would stop beside you offering you things… that if you wanted to sleep with them, they would take you where you wanted, or also that boys or older men would pass by and tell you, they would insult you by saying obscene things and well that scares me. (Junior high student, Group 3)

In addition to the crimes or aggressions that they directly experienced, events that were observed or talked about in the community also contributed to the participants feeling unsafe in the city. In the area surrounding the schools and on the students’ way home, sexual harassment was perpetrated primarily by adult men. Harassment is also carried out from the car, as they report feeling harassed when older strangers with sexual intentions follow them, block their path or invite them into the car, either to ask them to go out with them or offer them a lift. Additionally, the participants referred to theft of either personal possessions or school items. Armed robbery, especially the theft of money or cell phones when travelling between school and home, was mentioned to a greater extent by high school girls. Although they were mentioned less frequently, two situations that deserved attention were forced disappearances or attempted kidnappings.

We got out late from supper, about nine o’clock at night, so they wanted to pick up several girls in the year in which I was studying. It did not happen to me, but I heard about several cases. (University student, group 24)

They were chasing a friend recently and telling her things, dirty things. (High school student, Group 16)

In Mexico, the term non-located persons is often used for cases in which young women voluntarily run away from their homes. Reference is also made to missing persons, when they have a disability or mental health condition that prevents them from returning to their place of origin. The term forced disappearance is used mainly for those cases in which State agents participate directly in the disappearance of a person, or when the disappearance occurs with the complicity of the State. However, in recent decades the disappearance carried out by individuals without the participation of the State has increased. The disappearance most feared by young women is usually that related to individuals, especially with members of organized crime or people who exercise physical and sexual violence, commit feminicide and who subsequently disappear the bodies of the victims.

The participants perceived the neighbourhoods where they lived as more unsafe than the school since a greater variety of crimes was reported there and those crimes occurred more frequently and had greater impacts. In the neighbourhood, situations that also occurred in other spaces, such as robbery, especially of homes, robbery with the use of physical aggression or weapons, and sexual harassment were also common. The participants also described other worrisome events, such as homicides and forced disappearances.

Many times they just disappear. Several have already disappeared, and from this part [of town], from this area and from junior high school, they take them away, pick them up, rape them and then kill them. Or others, they are prostituting them on the black market—many things can happen—or they can no longer find them. They show up burned or all dismembered; many things can happen. (Junior high school student, group 8)

I went with two other cousins and they told us that they would give us 500 pesos if we got in the car, and then we told him no, and we kept walking… then he insisted, ‘Please get in’, and the other two were coming behind him. We answered, ‘We don’t want to’; we ran away, and the car was gone. (High school student, group 18)

**Fig 1:**
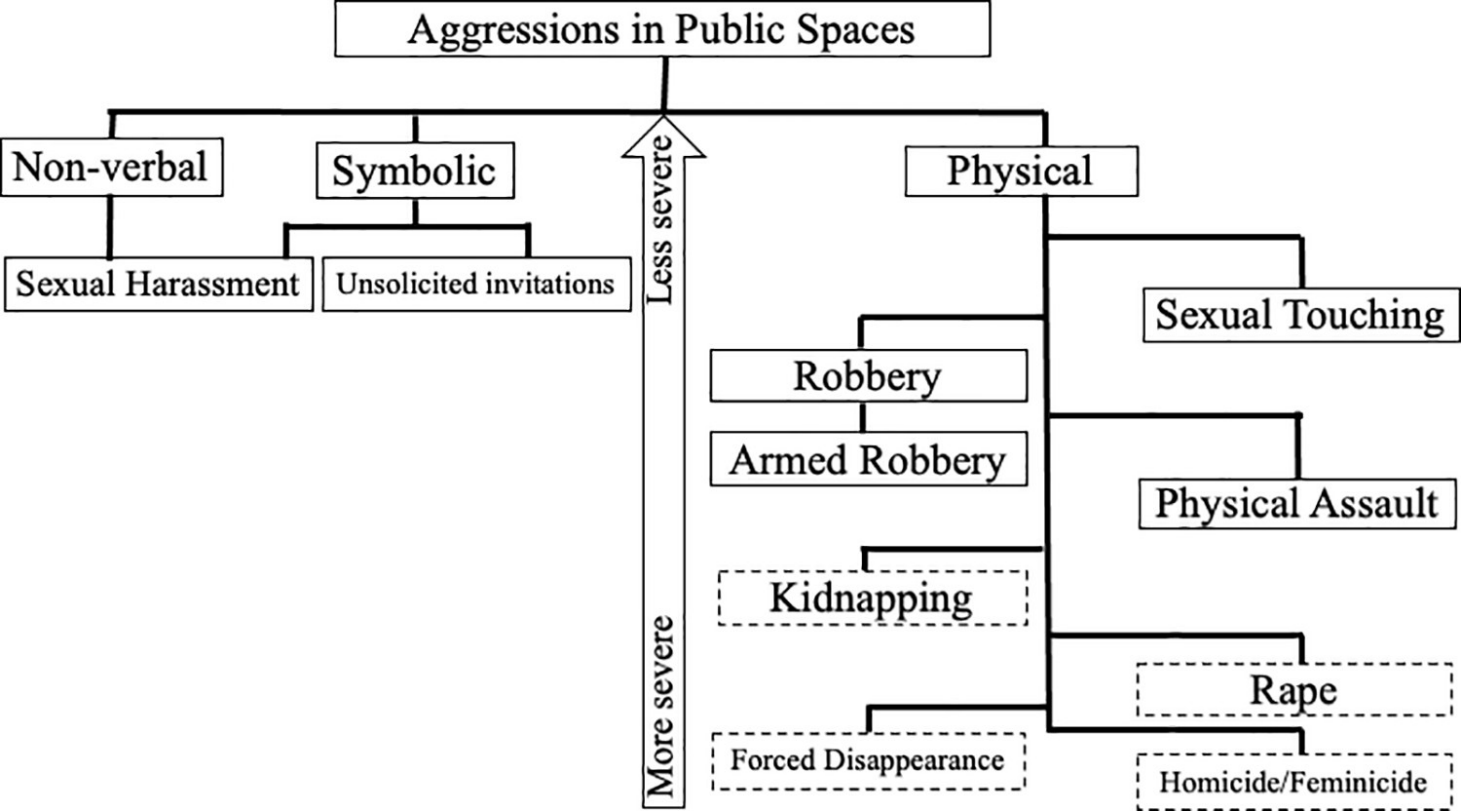
Aggressions in public spaces: School, neighbourhood and other areas of the city. Source: Own elaboration. Note 1: Sexual harassment was the only type of aggression that was also manifested nonverbally, primarily through leering, waiting, catcalling, exhibitionism, masturbating, and following. Note 2: Dashed lines indicate aggression experienced indirectly. Some participants suffered kidnapping attempts.

The aggressions that young women experience in public spaces (including transport services) can be grouped into non-verbal aggressions, symbolic aggressions and physical aggressions, which on a micro-interactional level can be considered violent because they involve kinetic contact. (Fig 1). Sexual harassment can be manifested verbally, non-verbally or through proximity without physical contact. These behaviors are performed by unknown men, and are experienced as unwelcome, one-way intrusions with sexual intent. Other symbolic or communicative aggressions are unsolicited invitations. Among the physical aggressions are the theft of objects or belongings, armed robbery or that resorts to the threat of physical aggression, as well as sexual touching. The aggressions mentioned previously are experienced both directly and indirectly. By indirect experience of the aggressions, it refers to observing or hearing that such acts occur, to close people such as family members, friends or classmates. Being close to a direct victim who has similar living conditions contributes to feeling vulnerable and affects their mobility in the city. Aggressions such as physical assault, kidnapping, rape, forced disappearance, homicide and feminicide are mainly experienced indirectly, although the attempted kidnapping was also experienced directly by some of the participants. They did not report having been raped in public space.

Aggressions are organized vertically in terms of severity, due to the fear they provoke, the degree of harm that is anticipated, and their psychosocial impact on daily life. At the pole of least severity are aggressions such as unsolicited invitations, sexual harassment, sexual touching, robbery and armed robbery. The young women experience these threats mainly directly, in spaces such as the neighborhood and the surroundings of the school. At the pole of greatest severity are aggressions such as physical assault, kidnapping, rape, forced disappearance, homicide and feminicide. These threats are mainly experienced indirectly, they can occur in their neighborhood, the school zone, but to a greater extent they are perceived as situations that characterize other places in the city, usually distant. These aggressions of greater severity can be related to organized crime activities. Aggressions such as sexual harassment and sexual touching have an impact that is amplified in daily life because when they experience it, young women anticipate becoming a victim of more severe aggression. Aggressions perceived as more severe are even interrelated. For example, kidnapping or enforced disappearance are associated with the possibility of physical assault, rape or even feminicide.

The severity continuum coincides with previous studies that indicate that physical aggression is perceived as more severe than psychological aggression [71, 72], in addition to presenting a greater correlation with physical deterioration [73]. In another study, it was found that people from the United States and Mexico ranked different types of aggression in a similar way, in terms of severity [74]. However, some studies indicate that compared to physical aggression, psychological aggression has a greater association with poor health [75], symptoms of psychological distress, anxiety or depression [76] or contributes to a greater degree to the development of post-traumatic stress disorder [77]. Physical aggression may be perceived as more severe because the damage is more visible and linked to a specific episode. Psychological aggression can be normalized because it occurs more frequently, although its impact can be continuous and cumulative [72].

The school and the neighbourhood were intimate spaces where young women travelled, established social ties and carried out their daily activities. However, the feeling of insecurity was also based on what happened in areas of the city where they did not usually go but have a mental representation of. Although robbery and armed robbery were present more frequently in those areas according to local reports [78], they were not perceived as the most problematic crimes. In these distant spaces, homicides, including feminicides, were perceived to occur more frequently, as do forced disappearances, especially of women and children, and, to a lesser extent, rapes. It is worth mentioning that with the exception of armed robbery, experiences of the various crimes were reported less frequently by junior high school girls than by other age groups.

Lately, I feel that this has happened more often, that I have heard at least three cases this year and perhaps at the end of last year… at least three that I have heard of girls who have had a boyfriend and who can’t be found or are found dismembered. One was found, I think, in neighbourhood A, in a house, where her romantic partner killed her. (High school student, group 11)

In several faculties, they were robbing girls and geez, how scary that you’re going to study and at some point you leave and they put you in a car and take you away. Even in several factories, several girls who work… my aunt had to witness a girl being grabbed and get pulled into a car and I think she managed to open the door and fell out… after a distance she fell out and managed to get away, but they already got on top of her (University student, Group 23).

Young women felt insecure because there were contextual conditions that exacerbated the feeling of a lack of protection (Table 2). Both the neighbourhoods where they lived and those that they perceived as even more dangerous were located in the areas with the highest crime incidence in the locality. These high-risk areas, mainly located on the periphery of the city, are also characterized by uneven urban development [79]. The perceived dangerousness of neighbourhoods was also constructed from daily conversations regarding the risk of using transit, the avoidance of certain spaces or the public discrediting of the sites.

**Table 2:** Contextual conditions of feelings of insecurity in public spaces: Main categories and subcategories across groups (n = 24).

Category	Subcategories	Frequency (%) ^a^
Threatening events in the school area	Sexual Harassment	12 (50.0%)
	Robbery	11 (45.8%)
	Armed Robbery	6 (25.0%)
	Forced Disappearance	5 (20.8%)
	Attempted Kidnapping	3 (12.5%)
Threatening events in the neighbourhood	Robbery	22 (91.6%)
	Armed Robbery	19 (79.2%)
	Sexual Harassment	15 (62.5%)
	Homicide and feminicide	15 (62.5%)
	Forced Disappearance	13 (54.2%)
Threatening events in other areas of the city	Homicide and feminicide	16 (66.6%)
	Forced Disappearance	16 (66.6%)
	Armed Robbery	12 (50.0%)
	Sexual Harassment	10 (41.6%)
	Rape	6 (25.0%)
	Robbery	4 (16.6%)
Characteristics of the neighbourhood	Sale of drugs	11 (45.8%)
	Drug use in public spaces	10 (41.6%)
	Shootings	9 (37.5%)
	Abandoned buildings	7 (29.2%)
	Vacant lots	6 (25.0%)
Situations of risk	Walking down the street	13 (54.2%)
	Going out at night	13 (54.2%)
	Travelling by bus	7 (29.2%)
	Walking alone	6 (25.0%)
Characteristics of the police	Absence	13 (54.2%)
	Corruption	9 (37.5%)
Characteristics of threatening actors	Age	
	Adult men	17 (70.8%)
	Young men	10 (41.6%)
	Appearance	
	“Tecolines”^b^	11 (45.8%)
	“Cholos”^c^	9 (37.5%)
	“Drug addicts”	7 (29.2%)

Source: Prepared by the authors.

^a^ The frequencies and percentages were obtained using MAXQDA 18 software, representing the number of groups in which each subcategory appeared.

^b^ The participants used ‘tecolines’ to describe people who have suffered physical, psychological and social deterioration due to substance use; they may live on the street, in abandoned houses or in traditional houses. They usually obtained some income from criminal activities, the sale of waste or merchandise and informal work.

^c^ They used ‘cholos’ to describe people who belong or have belonged to gangs, may engage in criminal activity or have withdrawn from it. They were characterized by a particular aesthetic in terms of hair (being shaved), clothing (wearing certain types of clothing, large sizes and specific brands) and having tattoos.

They were concerned about the sale of drugs on the street or in houses (they called ‘tienditas’ [‘little shops’] or ‘conectas’ [‘connections’] to the houses where illegal drugs were sold) because they perceived that conflicts might occur between groups linked to drug trafficking or that drug use would lead the occurrence of other common crimes, such as robbery or armed robbery. Increased drug use in neighbourhoods became a continuous threat for young women due to the fear that they would become victim to sexual crimes committed by consumers or that users would steal their possessions to maintain their addiction. The shootouts were another problem in the neighbourhood that worried them.

Other characteristics of the neighbourhoods were that they were isolated and uninhabited or had vacant lots, sites that have been associated with the discovery of the bodies of victims of feminicide [23]. Other factors were more situational, such as walking on the street, going out at night, walking alone and travelling by bus. The police also contributed to young women feeling unprotected [80] because of their absence in the communities or their corruption. The participants denounced the lack of surveillance in the neighbourhoods, the untimely arrival of the police and their lack of effectiveness in capturing the people who committed crimes.

Many times, the police even charge a fee. There, where I live, we all know it, but nobody says anything. There is a house where it is always full of people taking drugs; the police come by, they arrive, they stop, they collect what they have to pay, and they leave. (Junior high school student, group 8)

The young women revealed various characteristics of the actors who contributed to making them feel unsafe on the streets. They said that they were primarily older men, followed by young men. They mentioned that they were sexually harassed by men who travelled by car or who worked on the street. Although they indicated that unknown people were threatening, a general sense of threat predominated since the participants felt that anyone could assault them. They mentioned that they were mainly men perceived as “normal” or ordinary. In addition, the people perceived as a threat were menacing, socially marginalized men, whom the participants referred to as ‘tecolines’, ‘cholos’ or ‘drug addicts’:

When it happened to me, they were dressed normally. The first time he was young, not a man. He was dressed normally, and the boy was very handsome, but I ignored him, and it was when I ran out that he wanted to open the door to force himself on me. The second time it was an older man, who was also dressed like that with a long-sleeved button-down shirt. And the third time, it was a man who was also older, he was dressed in a suit. (High school student, Group 15)

It happened to me once, being left alone with a ‘tecolín’ and the driver, who also did not look very friendly. The three of us were in the front, and I went to the back. I said something, I changed the address, because I know the route of the bus. There was a door made of glass and a window; I threw myself [out]. No way—I would rather be beaten than be raped. (University student, group 24)

**Fig 2:**
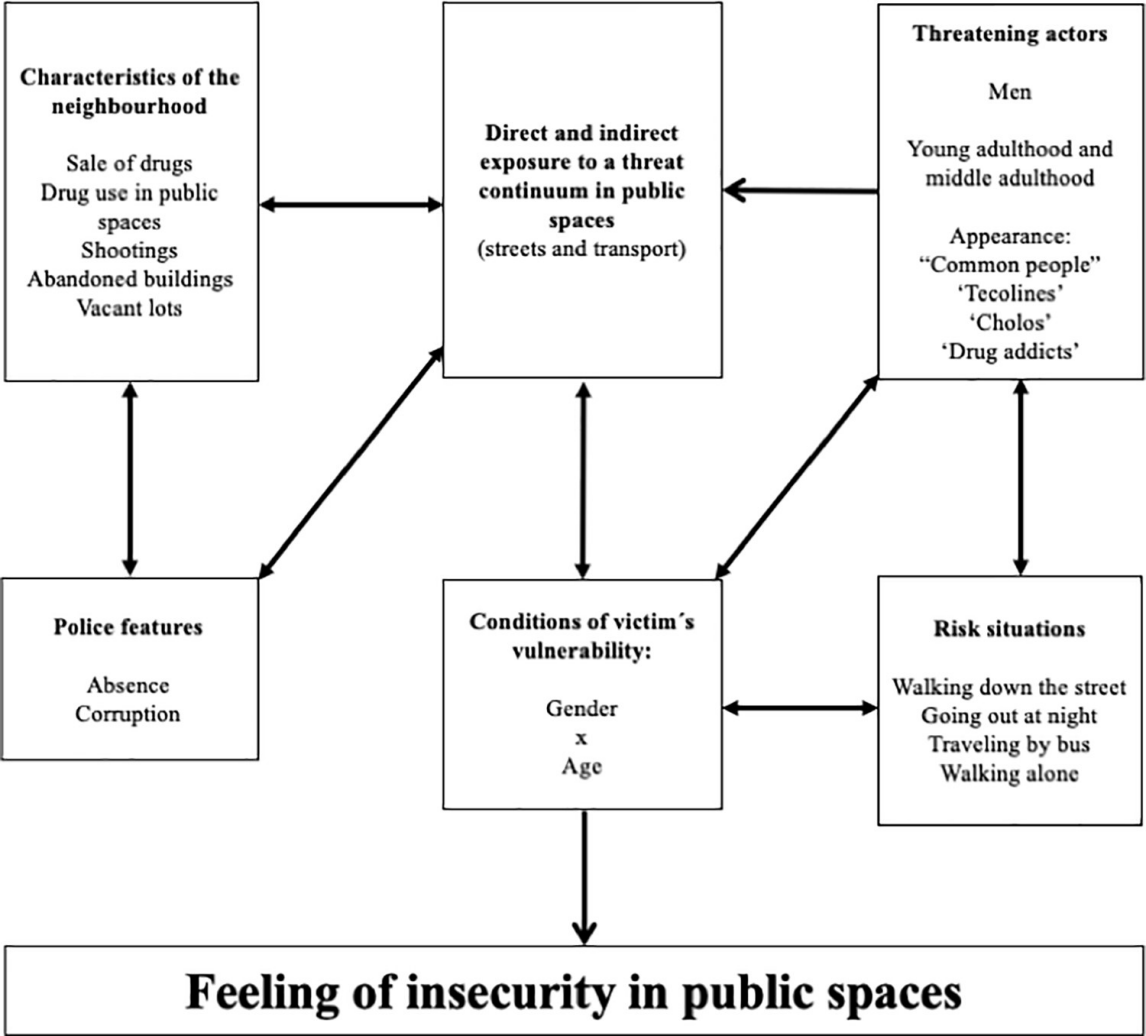
Contextual conditions of the feeling of insecurity in public spaces among young women. Source: Own elaboration.

The diverse contextual conditions favour the development of feelings of insecurity in a group that is vulnerable due to a combination of gender and age. Aggressions in public spaces occur due to the coexistence of situational risks, risky conditions in the neighbourhoods, the lack of protection figures or security in the streets and actors perceived as threatening (Fig 2).

### Feelings of insecurity in public spaces: Meanings and emotions

Insecurity had multiple meanings for the young women. First, it meant feeling that ‘something was going to happen to you’, that is, living with uncertainty and worry, especially when going out [1]. It had acquired a practical meaning because it implied a need to constantly look out for yourself. It also alluded to a characterization of space since it meant that these women lived in dangerous environments and that they had been exposed to crimes in their community [37].

Walking in fear that a stranger may do something to you. (Junior high school student, Group 4)

That you cannot leave your house for fear of being robbed. (High school student, Group 13)

It is to be in a constant state of alert, always looking around and thinking about what may happen to you, all possible situations. (University student, Group 21)

This disconnection from the security of previous life stages led to the emergence of multiple emotions in the young women. The central emotion was fear, as previously reported [1, 4]. At the same time, they also expressed ‘feeling bad’, which referred to a daily sense of general malaise that was not linked to a specific emotion. Other emotions expressed to a lesser extent were anger and sadness. ‘Feeling bad’ and sadness were reported mainly by the junior high school students. Some participants experienced insecurity as ‘something normal’, which can be considered an attempt to normalize everyday discomfort. This can become a form of affective anaesthetization, as a mechanism to deny or reduce negative emotions associated with insecurity and minimize concern about anticipated aggression, in order not to restrict mobility in public space.

I always think that a car is going to come and kidnap me and that they are going to do bad things to me, and it scares me. I am very paranoid, and I have always been afraid, and when they send me to the store, I ask my sister if she can come with me. (Junior high school student, group 6)

It’s next door to the store and we had to cross over to go to my house, so the guy was “like this” super slow, when we were already crossing and he stared at us a lot. And the truth is, it makes me very angry when they watch us, and I started to yell at him. It made me angry. (High school student, Group 20)

I don’t even feel comfortable going to the store, going to get the bus, because I have to go get the bus, as it kind of scares me. That is, leaving my house makes me cry. (University student, Group 24)

### Psychosocial consequences of the feeling of insecurity

Daily exposure to aggressions and criminal events and the existence of contextual conditions that limit their protection had various consequences in the lives of the young women. The main impact was a transformation in the way they experienced public space: when they went out, they lived with uncertainty, were concerned about possible risks and experienced distrust of others [81], which produced a constant need to be cautious.

There is a little corner near my house, and my mother tells me that they found a kidnapped woman there. So, when I go to get the bus; I pass by that house and I pass with a rush of fear, I walk quickly to my house. (High school student, Group 14)

Another consequence is the restriction on mobility. The young women stopped going out or limited their outings in terms of timing, the places they went, the way they travelled, or they needed to go out accompanied [82].

As you grow up, you realize how bad it is outside, and when you were a child, you were in the street as if nothing; you didn’t even know what it was to be robbed. And now, I cannot go there at night because they are going to rob me. (High school student, group 17)

One of the reasons why I am so behind this semester is precisely because of that, because of the bus, because it was dark and I told the teacher that I would rather be late than to be raped and killed or who knows what they would do to me. (University student, Group 23).

In a complementary way, there was an increase in parental protection, which coincided with the fact that parents had restricted their children’s outings due to the perception of a lack of safety in the state [78]. Parents developed various strategies to care for their young daughters, such as controlling giving them permission to go out, monitoring outings, accompanying them during transitions between locations, and teaching them precautionary measures or even negotiating with criminals in the neighbourhood to ensure that their daughters were not harmed. Parental protection was mentioned to a greater extent by junior high school girls than by participants in the other age groups and was gradually reduced as girls advanced in educational level:

My mom no longer lets me go out at night. I used to go out a lot at night, but she doesn’t let me go out anymore. There was a girl who was killed, I don’t remember, about three months ago, my mother knew her and so she didn’t let me go out at night. (Junior high school student, Group 2)

Before, they sent me a lot to the store and then news came out that a girl had been kidnapped and my mother wouldn’t let me go out alone. (Junior high school student, Group 5).

The young women reported interruptions in their recreational activities (Table 3). They stopped going to the park, playing sports on the street, going out with friends or visiting them in their homes; they stopped going to parties or clubs. Junior high school girls reported this interruption in their activities to a greater extent than the participants in other age groups. Finally, another consequence was a change of residence when family members decided to move due to the lack of safety, this implied changing school and the loss of social ties:

I also used to go for walks or train in boxing, but I can’t do it anymore because I changed schools, and if I go out more at night, I can’t because it is very unsafe for me. (High school student, group 19)

I live nearby and I kind of want to go by bike and my dad “no, you are not going to go by bike” because it is ugly out there. Fortunately, I have a car so the bike is no more. (University student, Group 22)

**Table 3:** Manifestations of feelings of insecurity in public spaces and its psychosocial consequences: Main categories and subcategories across groups (n = 24).

Categories	Subcategories	Frequency (%)
Meanings associated with insecurity	Feeling that something will happen	18 (75.0%)
	Having to take care of yourself	9 (37.5%)
	Exposure to crime	8 (33.3%)
	Inhabiting dangerous environments	8 (33.3%)
	Something normal	8(33.3%)
Emotions associated with insecurity	Fear	23 (95.8%)
	Discomfort	12 (50.0%)
	Anger	9 (37.5%)
	Sadness	8 (33.3%)
Strategies to manage the feeling of insecurity	*Relational*	
	Going out accompanied	18 (75.0%)
	Notifying family members about outings	9 (37.5%)
	Community relations	6 (25.0%)
	*Avoidance*	
	Changing appearance	14 (58.3%)
	Staying alert	13 (54.2%)
	Changing route	11 (45.8%)
	Changing outing times	9 (37.5%)
	Hiding belongings	8 (33.3%)
	Avoiding dangerous sites	8 (33.3%)
	*Defence*	
	Confrontation	10 (41.6%)
	Carrying weapons	9 (37.5%)
	*Internalize*	
	Don’t show fear	3 (12.5%)
	Don’t think about the risks	3 (12.5%)
Demands on the state regarding insecurity	Increased street surveillance	11 (45.8%)
	Improve police quality	10 (41.6%)
Psychosocial consequences of the feeling of insecurity	Transformation of the street experience	15 (62.5%)
	Restriction of mobility	13 (54.2%)
	Increased parental protection	13 (54.2%)
	Change of residence	8 (33.3%)
	Disruption of recreational activities	6 (25.0%)

Source: Prepared by the authors.

### Strategies for managing the feeling of insecurity

Starting in adolescence, women began to assume individual responsibility for their safety and developed a set of strategies to manage aggressions in the streets. In this stage, they initiated safety work, which is a set of adaptations, responses and practices to avoid crime or aggressions, reduce its escalation or refuse to participate in unwanted interactions.

The main strategy used was relational; they tried to go out accompanied by friends, colleagues or family [83], which was consistent with a process of socialization of fear of crime that promoted the search for safety through the presence of others in public spaces [10]. Another strategy was to inform family members about their outings, which involved providing information about the places they went to, the people they were with, their comings and goings and the approximate time they planned to return home. They used technology to share their location by mobile phone or to monitor the routes of private transport services. The last strategy consisted of maintaining relations with the community, which implied being known in their neighbourhood, establishing friendly relations with threatening figures and even providing them with economic support. The young university students did not report using the latter measure.

I always go with a male classmate; we take the same bus, and, well, I feel safe because, well, I’m already with a man, and if they say something to me, he can defend me. Another (female) classmate also goes with us, and, well, we are going ‘en bolita’ [in group]. If they do something to us, someone can do something or call the police. (High school student, group 19)

When I take an Uber, what I did on the way was I always called my boyfriend when he could not take me, it was like I called him for anything but I was talking to him so that he was tracking the car with his phone. (University student, Group 21)

The strategies young women used the most were aimed at avoiding the risk of aggressions [81, 83–85]. Of these strategies, the main one was changing their appearance, especially to prevent sexual harassment. When travelling on the street, they try to be alert, change routes and departure times, do not show belongings and avoid dangerous places. These measures also coincided with the socialization of fearing crime, which led to the restriction of mobility and changes in lifestyle as a form of adaptation [10] instead of using personal skills for self-defence and to obtain respect in the public space [85]. The high school girls resorted to changing their appearance and letting others know about their outings to a greater extent than the participants in other groups. Changes in departure times occurred more often among the junior high school girls and was used less as educational levels advanced.

If I want to go out in a dress or a skirt, I want to go out, and I cannot because they are going to yell things at you in the street, or they are going to take you to a vacant lot and rape you. (High school student, group 17)

Walking on different streets, changing routes, not having established routes and walking through different places, so that if someone is following me, they do not know where I am going to go. (Junior high school student, Group 9)

Another strategy used was to defend themselves against threatening actors. Women could verbally and, to a lesser extent, physically confront those who sexually harassed them or tried to rob them. It also involved taking photos of harassers to report them on social networks. Confrontation was used more often as the educational level increased. Some of the participants expressed walking with weapons, such as knives, pepper spray or devices that produce electric shocks [81]. The feeling of insecurity increased when young women distrusted the police and public order [35, 36]. Since urban mobility could not be completely restricted, young women were willing to use weapons in the hope that the state would provide protection or that the community would mobilize to demand security services in areas of the city that were not a priority for the state:

I always go out with a knife because they have already tried to assault me several times. (Junior high school student, group 8)

It has happened to me, seeing men who are watching girls, personally it has not happened to me here in the university, but I have seen men who are even following girls and the girls are in the group that we have here, in this faculty; they are very careful and describe the men, when it happens and how they do it, even taking photos of them. (University student, Group 22)

One last type of strategy adopted to a lesser extent is internalization, which implies a process of personal regulation to confront aggressions or to be able to move about in public space despite feeling threatened. Among these approaches are not showing fear and not thinking about risks.

A lot has happened to me, in the neighbourhood where I live; I ignore everything that surrounds me… I keep walking. (High school student, Group 20)

**Fig 3:**
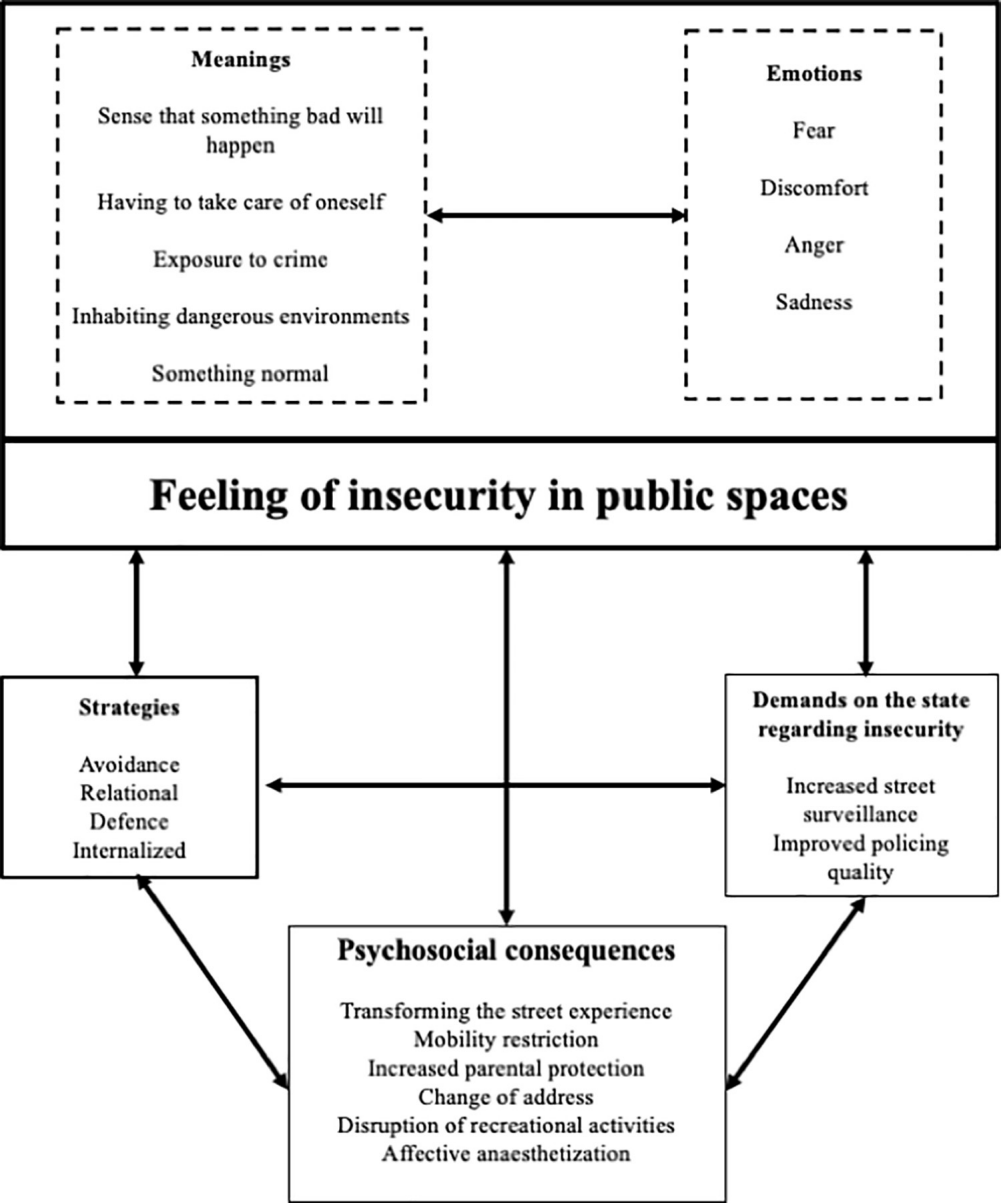
Components of the feeling of insecurity in public spaces and psychosocial consequences. Source: Own elaboration.

Young women proposed actions to address the lack of safety as a public problem since the feeling of insecurity also has political implications regarding the responsibility of the state to provide security (Fig 3). In the short term, they suggested that to reduce crime, greater vigilance was required on the streets; that the quality of policing must be improved by increasing the number of police, improving their training, and increasing their distribution throughout neighbourhoods; that crime prevention strategies must be developed; and that police must not fear confrontation with criminals. Reducing insecurity implied regaining trust in the police and monitoring areas where there has been uneven urban development:

It is necessary to add more security, such as patrols passing through and checking more. Yes, there are reports of many things in the ‘tienditas’ (‘little shops’), and the truth is that they do not do much. I know that it is not the only thing they have to do, but it is what they have to do, it is [the job of] the police. (Junior high school student, group 1).

## Discussion

This study shows the contextual conditions that influence the emergence of feelings of insecurity in public spaces by young women. Although this feeling partially arises from directly experiencing crimes with the highest incidence in the city, such as home robbery and armed robbery, adolescents and young adults also experience sexual harassment as an aggression that continually poses the threat of severe forms of physical aggression [86]. Sexual harassment is intentional, unwanted, not essential for survival and causes harm [87]. Sexual harassment is not usually carried out by a single individual repeatedly but is rather a systematic pattern of behaviour by unknown men. In a previous study, harassers indicated that the intention was mainly affective; they expected a positive response, and several of their behaviours could be interpreted as forms of dating [24]. However, since sexual harassment is an intrusive behaviour in the street by strangers with whom there is no relational history and an asymmetric dynamic when underage women are involved, it is not sufficient to dismiss their behaviour as courtship.

Sexual harassment in the streets is an expression of the sexual division of power; it represents a form of control of public space and an intensification of abuse due to the intersection of gender and age. At the same time, it generates fear that affects relationships with others and movement in space. It also supports social norms regarding the presentation of bodies and restricts when and where women can mobilize [88]. Young women are more vulnerable due to less ability to resist victimization or for being easier targets, in addition to being sought out to gratify the sexual desires of offenders [89].

The feeling of insecurity is based not only on direct victimization but also on becoming witnesses to aggressions against other women through either observing events or hearing stories during social interactions and in the media [10]. Although sexual harassment is an aggression that inaugurates a new way of experiencing mobility in the streets during adolescence [29], young women also feel vulnerable because of the possibility of experiencing crimes such as rape, forced disappearance and feminicide. Although rape has been considered a master offense in previous studies, it is possible that these offenses vary depending on the local context and that multiple interrelated master offenses coexist.

This study found that young women experience a continuum of threats on the street ranging from common crimes (robbery and armed robbery), daily forms of sexual aggression (sexual harassment and sexual touching) or acts experienced mainly indirectly (rape), and high-impact crimes that usually have a sexual nature (feminicide, forced disappearance or kidnapping). Young women experience a transformation of their experience in public spaces, even in cities that report low rates of violent crime. In the city of the study, robbery and armed robbery are the main crimes recorded; sexual harassment is not usually reported, and the homicide rate is low compared to other cities in the country [61]. Feminicides and forced disappearances are also underreported [62].

The feeling of insecurity among young women is not based solely on the incidence of crime or on the conversations and stories that record events that are not included in government reports on violence against women. It also arises from an imaginary dimension, since most of the participants expressed that the most severe aggressions occur mainly in areas of the city other than where they live or usually transit. According to cultivation theory, indirect exposure to aggressions against women in the media feeds a culture of fear. A distorted image of the world presented by the media is constructed that affects cognitive and affective states. In the stories presented, aggression against women is used to show norms and values, sending the message that the world is not safe and reinforcing stereotypes of women as vulnerable [90]. In the local context where the study was conducted, crimes such as feminicide or forced disappearance have particular relevance in print and electronic media but are also shared on social networks and in daily conversations.

Although this could also be interpreted as a psychological mechanism to feel more protected when travelling through public space, it is necessary to mention that the areas the participants named as representative hyperviolent neighbourhoods are those that consistently have high crime levels, are located on the periphery of the city or have limited access to public services, including security services [79]. Young women experience greater vulnerability to violent crimes if they have to travel alone, they do not have adequate protection networks or because their families cannot participate more in their daily care because they are working [91].

Forced disappearance especially feeds the imaginary dimension as it is characterized by uncertainty and is usually associated with other types of crimes. Disappearance implies movement, a transition from being a localized presence in a place to a disturbing lack of presence derived from a violent event, especially when this movement is involuntary and does not derive from a sense of agency [92]. In Mexico, the forced disappearance of women is linked to feminicide, and in public spaces, it is linked to a macho culture that makes women vulnerable to sexual aggressions as well as organized criminal violence. Women can suffer a double disappearance: when the state abandons them due to the stigma of violating gender norms associated with sexuality and drug use or conceives them as having less value than women in the upper classes or in favoured racial groups [93].

Young women’s fear of disappearance is also based on recent historical changes. Since 2008, the number of forced disappearances has increased in association with the government’s efforts against organized crime, reaching its highest level in 2017. The highest reports of loss and disappearance are among girls and young women aged 15 to 19 years, followed by women aged 20 to 24 years and girls aged 10 to 14 years. The association between the increase in forced disappearances of young women and trafficking for the purpose of sexual exploitation has been considered [94]. For trafficking, it has been found that older adolescents can be recruited by a romantic partner, while younger adolescents are usually recruited by acquaintances or strangers [95]. Although it is possible for victims to become trafficked through kidnapping, adolescent victims of human trafficking have indicated a more elaborate mechanism that involves deception and seduction [58]. Sexual harassment can provide an initial form of contact with the victim, especially in contexts where such harassment has been normalized and partnerships between adult men and adolescents have been legitimized.

The study was conducted in a border city in northern Mexico where drug trafficking has historically existed. It is worth mentioning that the participants did not make an explicit mention of drug trafficking, either due to attacks between criminal organizations or disputes with security authorities as has been reported in previous studies [96, 97]. In a minimal way, drug traffickers were mentioned as threatening figures. However, their presence is implicit in crimes partially linked to this type of criminal activity (such as feminicide and forced disappearance) and the sale and consumption of substances on public streets within their neighbourhoods when mentioning threatening figures involved in drug sales or who have deteriorated due to consumption, and in the fear of suffering some crime or aggressions for reasons associated with substance use.

The feeling of insecurity among young women can be observed from different angles. On the one hand, it indicates the transformation of their experience in the public space starting in adolescence. Changes in lifestyle have been reported due to the need to monitor the surroundings or having to go out in a group [24]. There is also the development of individual safety efforts [85] to protect themselves in contexts of vulnerability to aggressions against women, as has been found in previous studies [11]. The findings also reveal the maintenance of a structure of gender inequality that turns young women into sexual objects, restricts their mobility and autonomy [26] and causes them continuous emotional distress, mainly through fear and the threat of sexual violence, as in previous studies [25]. In contrast, other studies have reported the appearance of mental health problems such as anxiety, depression, self-objectification [24], posttraumatic stress disorder [15], behavioural vigilance and avoidance behaviours [14]. They need to generate strategies to take responsibility for their own safety, but most of these strategies indicate adjustment to this structure of gender inequality and the absence of protection mechanisms provided by the state. In the present study, strategies similar to those reported in previous studies were identified, such as avoidance [81, 84, 85], relational approaches [83], confrontation [85, 98], the use of weapons [81] and reporting online [85].

The findings also coincide with strategies for coping with intrusive relational obsession and stalking, such as moving outward, moving away, moving against and moving inward [99]. No evidence was found of the use of the moving with strategy [100], which consists of trying to negotiate or persuade the threatening actor to recognize the victim’s discomfort or preferences and desist from their unwanted activity. Young women may prefer to use other strategies due to the anticipation of more severe aggressions, and the perception that they cannot negotiate or persuade threatening figures, in a relationship where there is an imbalance of power.

Previous studies found differences between the perceived crimes as more frequent and the perceived crimes as more feared, due to their potential damage to physical integrity. Sexual crimes are more feared even though they are not seen as more frequent, especially when participants are adult persons of a greater age range [51, 101]. The present study shows the relevance to perform diagnosis with vulnerable groups like young women, since it reveals a different configuration of threats in the public space, it emphasizes the importance of sexual harassment in everyday life and the anticipation of violent crimes.

In previous studies, it was also found that the perception of insecurity is related to signs of physical disorganization or physical incivilities, the presence of the police and direct victimization [5, 51]. In another study, it was identified that the indicators of insecurity depend on the context in which the diagnosis is made, since there are places where the experience of direct and indirect victimization is a central element, while in other places non-criminal aspects such as social disorganization and trust in the police exert greater influence. In the same study, gender and trust in others were found to persist across contexts [52]. In the place where the study was carried out, these elements were also reported by the participants, although it is worth noting the degree of influence of indirect victimization in anticipating violent crimes and the mistrust they experience in public space, especially towards men.

The study findings partially agree with a model where feelings of insecurity are associated with signs of physical and social impairment, perceived risk of victimization, fear of personal harm, while security is favored by trust in the police [53]. Among the differences is that young women express emotions other than fear and the influence of anxiety as a trait is not explored in the present study. There is greater concern about personal harm compared to theft of property, which may be due to the fact that at this stage of life, most young people depend financially on their parents. Young women did not consider the importance of collective efficacy as an element that provides security. This may be related to less active participation in community life and a more passive stance regarding public security, since the State is expected to increase vigilance and improve the quality of the police. Other differences lie in the fact that this study promotes the incorporation of other elements to understand insecurity, such as the victim’s vulnerability conditions, risk situations, psychosocial consequences, and strategies to deal with threats.

The concept of feeling of insecurity has been used in previous studies to understand the experience of adult populations in contexts of high violence exerted by organized crime [96, 97]. This category is useful to consider the experience of young women and adolescents, although it is important to take into account the following aspects. The situations associated with feelings of insecurity not only are crimes but can also include aggressions not classified as crimes. These situations can occur objectively or be experienced indirectly, especially in the case of contemporary perceptual offenses. The feeling of insecurity is composed of an interpretive framework where meanings and emotions are interrelated, which in turn interact with security strategies. Although the public position regarding the problem is latent, public participation regarding this social problem is less common. Likewise, it is necessary to consider that the intersection between gender and age creates particular conditions of vulnerability that expose young women to the continuum of threats in the public space, amplifies their impact on daily life and limits the agency for their control.

Finally, it is worth noting that the participants’ demands revolve around surveillance in the streets and improvement in policing, which is an indicator of the sense of vulnerability and the urgent need for short-term measures. Taking into account the theory of routine activities [102], this study revealed the security efforts carried out by young women to avoid becoming victims of multiple aggressions, in addition to the role of guardians carried out by their families and friends, either through accompaniment when travelling through the city or monitoring their activities through electronic devices. However, it would be important to promote the participation of other guardians, such as police and community members, considering the mobile nature of the threats on the street.

Prevention policies that aim to reduce the normalization of sexual harassment on the street, the silencing of reports of sexual harassment and victim-blaming are recommended. It is necessary to facilitate the mobility of young women in the city by improving access to public transportation and security services, especially in places where rapid urbanization and poor planning have favoured the development of areas with poor infrastructure and social segregation. Likewise, it is important to create institutional surveillance mechanisms of gender violence, facilitate reporting by young women and the joint development of programmes where communities link with government agents to create secure environmental conditions. Such programmes require incorporation of the voices and perspectives of young women.

Future studies may include the perspective of young women belonging to other socioeconomic levels, since the participants belonged mainly to the middle socioeconomic level. It would also be important to include the perspective of young people who attend private schools and young people who are not in school. It should be remembered that this study takes place in a city on the border in the northern region of Mexico and that it is necessary to contrast the findings with cities that present different conditions of urban security for women. Additionally, while many threats in the public space are of a sexual nature in the context of heterosexuality, young women can also suffer threats such as discrimination and physical and verbal hostility [103], classified as racist, homophobic or transphobic [11].

## Supporting information

S1 Appendix (DOCX)

## References

[1] Cops D. Socializing into fear. The impact of socializing institutions on adolescents´ fear of crime. Young. 2010; 18(4): 385–402. doi: 10.1177/110330881001800402

[2] Ferraro K F. Fear of crime: interpreting victimization risk. 1st ed. New York: State University of New York Press; 1995.

[3] Grinshteyn EG, Eisenman DP, Cunningham WE, Andersen R, Ettner SL. Individual- and neighborhood-level determinants of fear of violent crime among adolescents. Fam Community Health. 2016; 39(2): 103–112. doi: 10.1097/FCH.0000000000000095 26882413

[4] Reyes V, Reséndiz A, Alcázar RJ, Reidl LM. [Coping strategies assumed by teenagers in situations that cause fear]. Psicogente. 2017; 20(38): 240–255. doi: 10.17081/psico.20.38.2544 Spanish

[5] Collins RE. Addressing the inconsistencies in fear of crime research: A meta-analytic review. J Crim Justice. 2016; 47: 21–31. doi: 10.1016/j.jcrimjus.2016.06.004

[6] Özaşçılar M, Ziyalar N. Unraveling the determinants of fear of crime among men and women in Istanbul: Examining the impact of perceived risk and fear of sexual assault. Int J Offender Ther Comp Criminol. 2017; 61(9): 993–1010. doi: 10.1177/0306624X15613334 26515412

[7] Rader NE, Cossman JS, Porter JR. Fear of crime and vulnerability: Using a national sample of Americans to examine two competing paradigms. J Crim Justice. 2012; 40(2): 134–141. doi: 10.1016/j.jcrimjus.2012.02.003

[8] Snedker KA. Explaining the gender gap in fear of crime: Assessments of risk and vulnerability among New York City residents. Fem Criminol. 2012; 7(2): 75–111. doi: 10.1177/1557085111424405

[9] Jackson J. A psychological perspective on vulnerability in the fear of crime. Psychol Crime Law. 2009; 15(4): 365–390. doi: 10.1080/10683160802275797

[10] Rader N, Haynes S. Gendered fear of crime socialization: an extension of Aker´s social learning theory. Fem Criminol. 2011; 6(4): 291–307. doi: 10.1177/1557085111408278

[11] Fileborn B, O’Neill T. From “ghettoization” to a field of its own: a comprehensive review of street harassment research. Trauma Violence Abuse. 2021; 15248380211021608. doi: 10.1177/15248380211021608 34098825

[12] May DC, Rader NE, Goodrum S. A gendered assessment of the “Threat of Victimization”: Examining gender differences in fear of crime, perceived risk, avoidance, and defensive behaviors. Crim Justice Rev. 2010; 35(2): 159–182. doi: 10.1177/0734016809349166

[13] Senn CY, Dzinas K. Measuring fear of rape. Can J Behav Sci. 1996; 28: 141–144. doi: 10.1037/0008-400x.28.2.141

[14] McDonald MM, Coleman B, Brindley S. Calibrating fear of rape: Threat likelihood and victimization costs. Pers Individ Dif. 2019; 139: 326–330. doi: 10.1016/j.paid.2018.12.001

[15] Spohn R, Wright EM, Peterson JC. Rape and mental health outcomes among women: Examining the moderating effects of “healthy” fear levels. Violence Against Women. 2017; 23(9): 1055–1075. doi: 10.1177/1077801216655625 27378718

[16] Hilinski CM. The role of victim-offender relationships in predicting fear of rape among college women. Crim Justice Stud. 2010; 23(2): 147–162. doi: 10.1080/1478601X.2010.485477

[17] Britto S, Stoddart D, Ugwu J. Perceptually contemporaneous offenses: Gender and fear of crime among African-American university students. J Ethn Crim Justice. 2018; 16(2): 117–136. doi: 10.1080/15377938.2017.1354117

[18] Özascilar M. Predicting fear of crime: A test of the shadow of sexual assault hypothesis. Int Rev Vict. 2013; 19(3): 269–284. doi: 10.1177/0269758013492754

[19] United Nations Office on Drugs and Crime. Global study on homicide 2018. Gender-related killing of women and girls [Internet]. Vienna: United Nations Office on Drugs and Crime; 2018 [cited 2020 Apr 6]. Available from: https://www.unodc.org/documents/data-and-analysis/GSH2018/GSH18_Gender-related_killing_of_women_and_girls.pdf

[20] Castañeda MP. Feminicide in Mexico: an approach through academic, activist and artistic work. Curr Sociol. 2016; 64(7): 1054–1070. doi: 10.1177/0011392116637894

[21] Monárrez JE, Cervera LE. Actualización y georreferenciación del feminicidio en Ciudad Juárez (1993–2010) [Updating and georeferencing of feminicide in Ciudad Juárez (1993–2010)]. In: Cervera L J. Monárrez J, coordinators. Geografía de la violencia en Ciudad Juárez, Chihuahua [Geography of violence in Ciudad Juárez, Chihuahua]. Tijuana: El Colegio de la Frontera Norte; 2013. pp. 63–100. Spanish.

[22] Mbembe A. Necropolítica [Necropolitics]. Madrid: Melusina; 2011. Spanish.

[23] Monárrez JE. Feminicidio: Muertes públicas, comunidades cerradas y estado desarticulado [Feminicide: public deaths, closed communities, and a disjointed state]. In: Monárrez J, Robles R, Cervera L J., Fuentes C, coordinators. Vidas y territorios en busca de justicia [Lives and territories in search of justice]. Tijuana: El Colegio de la Frontera Norte y Universidad Autónoma de Ciudad Juárez; 2015. pp. 109–149. Spanish.

[24] DelGreco M, Ebesu Hubbard AS, Denes A. Communicating by catcalling: Power dynamics and communicative motivations in street harassment. Violence Against Women. 2021; 27(9): 1402–1426. doi: 10.1177/1077801220927085 32567540

[25] Betts L, Harding R, Peart S, Sjolin Knight C, Wright D, Newbold K. Adolescents’ experiences of street harassment: Creating a typology and assessing the emotional impact. J Aggress Confl Peace Res. 2019; 11(1): 38–46. doi: 10.1108/JACPR-12-2017-0336

[26] Bastomski S, Smith P. Gender, fear, and public places: How negative encounters with strangers harm women. Sex Roles. 2017; 76(1–2): 73–88. doi: 10.1007/s11199-016-0654-6

[27] Bailey B. Greetings and compliments or street harassment? Competing evaluations of street remarks in a recorded collection. Discourse Soc. 2017; 28(4): 353–373. doi: 10.1177/0957926517702979

[28] Spitzberg BH. Acknowledgment of unwanted pursuit, threats, assault, and stalking in a college population. Psychol Violence. 2017; 7(2): 265–275. doi: 10.1037/a0040205

[29] Johansson K, Laflamme L, Eliasson M. Adolescents´ perceived safety and security in public space–a swedish focus group study with a gender perspective. Young. 2012; 20(1): 69–88. doi: 10.1177/110330881102000104

[30] Meza-de-Luna ME, García-Falconi S. Adolescent street harassment in Querétaro, Mexico. Affilia. 2015; 30(2): 158–169. doi: 10.1177/0886109914541117

[31] Spitzberg BH. Sexual coercion in courtship relations. In Spitzberg BH, Cupach WR, editors. The dark side of close relationships. New Jersey: Erlbaum; 1998. pp. 179–232.

[32] Lenton R, Smith MD, Fox J, Morra N. Sexual harassment in public places: Experiences of Canadian women. Can Rev Sociol Anthropol. 1999; 36(4): 517–540. doi: 10.1111/j.1755-618X.1999.tb00962.x

[33] Gardner CB. Passing by: Street remarks, address rights, and the urban female. Sociol Inq. 1980; 50(3/4): 328–356. doi: 10.1111/j.1475-682X.1980.tb00026.x

[34] Vieno A, Roccato M, Russo S. Is fear of crime mainly social and economic insecurity in disguise? A multilevel multinational analysis. J Community Appl Soc Psychol. 2013; 23(6): 519–535. doi: 10.1002/casp.2150

[35] Gaitán-Rossi P, Shen C. Fear of crime in Mexico: the impacts of municipality characteristics. Soc Indic Res. 2018; 135(1): 373–399. doi: 10.1007/s11205-016-1488-x

[36] Molina-Coloma V, Reyes-Sosa H, Larrañaga-Egilegor M. [The social representation of insecurity in young ecuadorians: the Ambato case]. Pensando Psicol. 2015; 11(18): 85–95. doi: 10.16925/pe.v11i18.1221 Spanish.

[37] Brunton-Smith I. Untangling the relationship between fear of crime and perceptions of disorder: evidence from a longitudinal study of young people in England and Wales. Br J Criminol. 2011; 51(6): 885–899. doi: 10.1093/bjc/azr064

[38] Killias M, Clerici C. Different measures of vulnerability in their relation to different dimensions of fear of crime. Br J Criminol. 2000; 40(3): 437–450. doi: 10.1093/bjc/40.3.437

[39] Kohm SA. Spatial dimensions of fear in a high-crime community: Fear of crime or fear of disorder? Can J Criminol. 2009; 51(1): 1–30. doi: 10.3138/cjccj.51.1.1

[40] Shechory-Bitton M, Cohen-Louck K. Does fear of terrorism differ from fear of crime and sexual assault: A question of geographical location and residential area. Int J Offender Ther Comp Criminol. 2018; 62(3): 806–826. doi: 10.1177/0306624X16658472 27383073

[41] Pearson AL, Breetzke GD. The association between the fear of crime, and mental and physical wellbeing in New Zealand. Soc Indic Res. 2013; 119(1): 281–294. doi: 10.1007/s11205-013-0489-2

[42] Villareal A, Yu W. Crime, fear and mental health in Mexico. Criminology. 2017; 55(4): 779–805. doi: 10.1111/1745-9125.12150

[43] Ávila ME, Martínez B, Vera A, Bahena A, Musitu G. [Victimización, percepción de inseguridad y cambios en las rutinas cotidianas en México]. Rev Saude Publica. 2016; 50(60): 1–9. doi: 10.1590/S1518-8787.2016050006098 Spanish 27706373 PMC5068965

[44] Martínez-Ferrer B, Ávila-Guerrero ME, Vera-Jiménez JA, Bahena-Rivera A, Musitu-Ochoa G. [Satisfacción con la vida, victimización y percepción de inseguridad en Morelos, México]. Salud Publica Mex. 2016; 58(1): 16–24. Available from: https://www.medigraphic.com/cgi-bin/new/resumen.cgi?IDARTICULO=65497. Spanish. 26879503

[45] Lorenc T, Clayton S, Neary D, Whitehead M, Petticrew M Thomson H, et al. Crime, fear of crime, environment, and mental health and wellbeing: mapping review of theories and causal pathways. Health Place. 2012; 18(4): 757–765. doi: 10.1016/j.healthplace.2012.04.001 22542441

[46] Gray E, Jackson J, Farrall S. Feelings and functions in the fear of crime. Br J Criminol. 2011; 51: 75–94. doi: 10.1093/bjc/azq066

[47] Jackson J, Gray E. Functional fear and public insecurities about crime. Br J Criminol. 2010; 50(1): 1–22. doi: 10.1093/bjc/azp059

[48] Kessler G. [Algunas hipótesis sobre la extensión del sentimiento de inseguridad en América Latina]. Cuad Antropol Soc. 2013; 37: 25–42. Available from: http://sedici.unlp.edu.ar/handle/10915/111039. Spanish.

[49] Lupton D, Tulloch J. Theorizing fear of crime: beyond the rational/irrational opposition. Br J Sociol. 1999; 50(3): 507–523. doi: 10.1111/j.1468-4446.1999.00507.x 15259198

[50] Béland D. Insecurity and politics: a framework. Can J Sociol. 2007; 32(3): 317–340. doi: 10.2307/20460646

[51] Azevedo V, Sani A, Nunes LM, Paulo D. Do you feel safe in the urban space? From perceptions to associated variables. Anu Psicol Jurid. 2021; 31(1): 75–84. doi: 10.5093/apj2021a12

[52] Valente R, Vacchiano M. Determinants of the fear of crime in Argentina and Brazil: a cross-country comparison of non-criminal and environmental factors affecting feelings of insecurity. Soc Indic Res. 2021; 154(3): 1077–1096. doi: 10.1007/s11205-020-02545-y

[53] Reid ID, Appleby-Arnold S, Brockdorff N, Jakovljev I, Zdravković S. Developing a model of perceptions of security and insecurity in the context of crime. Psychiatry Psychol Law. 2020; 27(4): 620–636. doi: 10.1080/13218719.2020.1742235 33679201 PMC7901684

[54] Fernandes L, Rêgo X. [Where is the feeling of insecurity? Social and scientific problematizations of fear of the city]. Etnografica. 2011; 15(1): 167–181. doi: 10.4000/etnografica.869 Portuguese.

[55] Luneke A. [Urban insecurity, citizen participation, and neighboring care: the quest for protection in neighborhoods]. Rev INVI. 2021; 36(102): 302–327. doi: 10.4067/S0718-83582021000200302 Spanish.

[56] de Mattos M. [Power relations and social control in violent areas]. Civ Rev Cienc Sociais. 2015; 15(1): 84–104. doi: 10.15448/1984-7289.2015.1.16934 Portuguese.

[57] Kessler G. El sentimiento de inseguridad. Sociología del temor al delito [The feeling of insecurity. Sociology of fear of crime]. Buenos Aires: Siglo XXI editors; 2011. 287 p. Spanish.

[58] Rocha T, Brouwer KC, Salazar M, Boyce SC, Servin AE, Goldenberg SM, et al. “He invited me and didn´t ask anything in return” Migration and mobility as vulnerabilities for sexual exploitation among female adolescents in Mexico. Int Migr. 2018; 56(2): 5–17. doi: 10.1111/imig.12333 33293733 PMC7720900

[59] Instituto Nacional de Estadística y Geografía. Encuesta nacional de seguridad pública urbana ENSU Tercer trimestre 2019. Principales resultados [National Survey of Urban Public Safety ENSU Third quarter 2019. Main results]. [Internet]. Aguascalientes: INEGI; 2019 [cited 2020 Mar 6]. Available from: https://www.inegi.org.mx/programas/ensu/. Spanish.

[60] Incidencia delictiva estatal [State crime incidence] [Internet]. Mexicali: Guardia Estatal de Seguridad e Investigación. c2018 –[2018 Apr 30]. Available from: https://www.seguridadbc.gob.mx/contenidos/ESTADISTICAS.php. Spanish.

[61] Incidencia delictiva del fuero común, nueva metodología [Criminal incidence of the common jurisdiction, new methodology] [Internet]. Ciudad de México: Secretariado Ejecutivo del Sistema Nacional de Seguridad Pública. c2020 –[cited 2020 Mar 16]. Available from: https://www.gob.mx/sesnsp/acciones-y-programas/incidencia-delictiva-del-fuero-comun-nueva-metodologia. Spanish.

[62] Haro S. La vida en rosa… las muertas de Mexicali [Life in pink… the dead women of Mexicali]. Mexicali: Universidad Autónoma de Baja California; 2017. Spanish.

[63] Reconstrucción de nombres de personas desaparecidas [Reconstruction of names of disappeared persons] [Internet]. Ciudad de México: Data Cívica. c2020 –[cited 2020 Mar 13]. Available from: http://personasdesaparecidas.mx/db/db. Spanish.

[64] Charmaz K. Constructing Grounded Theory. 1st ed. California: Sage; 2006.

[65] AMAI. Cuestionario NSE AMAI 2018 [SES AMAI 2018 Questionnaire] [Internet]. Ciudad de México: AMAI; 2018 [cited 2018 Mar 14]. Available from: https://nse.amai.org/cuestionarios/. Spanish.

[66] Charmaz K, Thornberg R. The pursuit of quality in grounded theory. Qual Res Psychol. 2021; 18(3): 305–327. doi: 10.1080/14780887.2020.1780357

[67] Charmaz K. Teaching theory construction with initial grounded theory tools: A reflection on lessons and learning. Qual Health Res. 2015; 25(12): 1610–1622. doi: 10.1177/1049732315613982 26646825

[68] Andersen P, Inoue K, Walsh K. An animated model for facilitating understanding of Grounded Theory and the processes used to generate substantive theory. J Res Nurs. 2013; 18(8): 734–743. doi: 10.1177/1744987111434188

[69] Chiovitti RF, Piran N. Rigour and grounded theory research. J Adv Nurs. 2003; 44(4): 427–435. doi: 10.1046/j.0309-2402.2003.02822.x 14651715

[70] Instituto Nacional de Estadística y Geografía. Encuesta nacional sobre la dinámica de las relaciones en los hogares ENDIREH 2016 [National survey on the dynamics of household relationships ENDIREH 2016] [Internet]. Aguascalientes: INEGI; 2017 [cited 2020 Mar 13]. Available from: https://www.inegi.org.mx/programas/endireh/2016/. Spanish.

[71] Wilson JM, Smirles K. College students’ perceptions of intimate partner violence: The effects of type of abuse and perpetrator gender. J Interpers Violence. 2022; 37(1–2): 172–194. doi: 10.1177/0886260520908025 32125215

[72] Capezza NM, Arriaga XB. You can degrade but you can’t hit: Differences in perceptions of psychological versus physical aggression. J Soc Pers Relat. 2008; 25(2): 225–245. doi: 10.1177/0265407507087957

[73] Fernández-Fuertes AA, Fuertes A. Physical and psychological aggression in dating relationships of Spanish adolescents: Motives and consequences. Child Abuse Negl. 2010; 34(3): 183–191. doi: 10.1016/j.chiabu.2010.01.002 20207002

[74] Peek-Asa C, Garcia L, McArthur D, Castro R. Severity of intimate partner abuse indicators as perceived by women in Mexico and the United States. Women Health. 2002; 35(2–3): 165–180. doi: 10.1300/J013v35n02_11 12201506

[75] Dichter ME, Marcus SC, Wagner C, Bonomi AE. Associations between psychological, physical, and sexual intimate partner violence and health outcomes among women veteran VA patients. Soc Work Ment Health. 2014; 12(5–6): 411–428. doi: 10.1080/15332985.2013.870104

[76] Jouriles EN, Garrido E, Rosenfield D, McDonald R. Experiences of psychological and physical aggression in adolescent romantic relationships: Links to psychological distress. Child Abuse Negl. 2009; 33(7): 451–460. doi: 10.1016/j.chiabu.2008.11.005 19589597 PMC3951513

[77] Pico-Alfonso MA. Psychological intimate partner violence: The major predictor of posttraumatic stress disorder in abused women. Neurosci Biobehav Rev. 2005; 29(1): 181–193. doi: 10.1016/j.neubiorev.2004.08.010 15652265

[78] Instituto Nacional de Estadística y Geografía. Encuesta nacional de victimización y percepción sobre seguridad pública ENVIPE 2019. Principales resultados, Baja California [National survey of victimization and perception of public safety ENVIPE 2019. Main results, Baja California] [Internet]. Aguascalientes: INEGI; 2019 [cited 2020 Mar 13]. Available from: https://www.inegi.org.mx/programas/envipe/2019/. Spanish.

[79] González F. Geografía y violencia. Una aproximación conceptual al fundamento espacial de la violencia estructural [Geography and violence. A conceptual approach to the spatial foundation of structural violence]. Ciudad de México: Ediciones Monosílabo; 2018. Spanish.

[80] Martínez-Ferrer B, Vera A, Musitu G, Montero-Montero D. Trust in police and fear of crime among young people from a gender perspective: the case of Mexico. Violence Gend. 2018; 5(4): 1–7. doi: 10.1089/vio.2017.0080

[81] Campos PA, Falb KL, Hernández S, Díaz-Olavarrieta C, Gupta J. Experiences of street harassment and associations with perceptions of social cohesion among women in Mexico City. Salud Publica Mex. 2017; 59(1): 102–105. doi: 10.21149/7961 28423116

[82] Vera A, Ávila M, Martínez-Ferrer B, Musitu G, Montero D. [Perception of insecurity, victimization and restrictions in daily life according to the life cycle, in Morelos, Mexico]. Rev Crim. 2017; 59(3): 183–192. Available from: http://www.scielo.org.co/scielo.php?pid=S1794-31082017000300183&script=sci_abstract&tlng=en. Spanish.

[83] Alcalde C. Gender, autonomy and return migration: negotiating street harassment in Lima, Peru. Glob Netw. 2018; 20(1): 25–41. doi: 10.1111/glob.12218

[84] Cortazar FJ. [Sexual harassment at University of Guadalajara. The students speak]. La Ventana. 2019; 50(2): 175–204. Available from: http://www.scielo.org.mx/scielo.php?pid=S1405-94362019000200175&script=sci_abstract&tlng=en. Spanish.

[85] Fleetwood J. Everyday self-defence: Hollaback narratives, habitus and resisting street harassment. Br J Sociol. 2019; 70(5): 1709–1729. doi: 10.1111/1468-4446.12699 31402455

[86] Lennox R, Jurdi-Hage R. Beyond the empirical and the discursive: the methodological implications of critical realism for street harassment research. Womens Stud Int Forum. 2017; 60(1): 28–38. doi: 10.1016/j.wsif.2016.11.010

[87] Hamby S. On defining violence, and why it matters. Psychol Violence. 2017; 7(2): 167–180. doi: 10.1037/vio0000117

[88] Connell RW. Masculinities. 1st ed. California: University of California Press; 1995.

[89] Finkelhor D, Asdigian NL. Risk factors for youth victimization: beyond a lifestyles/routine activities theory approach. Violence Vict. 1996; 11(1): 3–19. Available from: http://unh.edu/ccrc/pdf/CV13.pdf 8870212

[90] Custers K, Van den Bulck J. The cultivation of fear of sexual violence in women: Processes and moderators of the relationship between television and fear. Communic Res. 2013; 40(1): 96–124. doi: 10.1177/0093650212440444

[91] Lozano-Reich NM. Reconceptualizing feminicidio: border materiality in Ciudad Juárez. Womens Stud Commun. 2018; 41(2): 104–107. doi: 10.1080/07491409.2018.1463767

[92] Stevenson O, Parr H, Woolnough P. Missing women: policing absence. Trans Inst Br Geogr. 2017; 42(2): 220–232. doi: 10.1111/tran.12160

[93] Binkowski B. Mexico´s epidemic of missing and murdered women. The Globe and Mail. 2015 July 12 [Cited 2020 April 14]. Available from: https://www.theglobeandmail.com/news/world/mexicos-epidemic-of-missing-and-murdered-women/article25137141/

[94] Ortiz A. Missing women: mexican female teenagers are most likely to dissappear. El Universal. 2020 Mar 3 [Cited 2020 April 10]. Available from: https://www.eluniversal.com.mx/english/missing-women-mexican-female-teenagers-are-most-likely-disappear

[95] Rocha T, Salazar M, Boyce SC, Brouwer KC, Staines H, Silverman JG. “We were isolated and we had to do whatever they said”: violence and coercion to keep adolescents girls from leaving the sex trade in two U.S.-Mexico border cities. J Hum Traffick. 2018; 5(4): 312–324. doi: 10.1080/23322705.2018.1519753

[96] Gómez AH, Almanza AM. [Impact of drug trafficking in young adults from Tamaulipas, Mexico: drugs and insecurity]. Rev Psicol. 2016; 34(2): 445–472. Spanish. doi: 10.18800/psico.201602.009 Spanish.

[97] Almanza AM, Romero MP, Gómez AH. Feelings of security regarding organized crime in Tamaulipas, Mexico. Salud Publica Mex. 2018; 6(4): 442–450. doi: 10.21149/8087 30137946

[98] Vera-Gray F. Men´s stranger intrusions: rethinking street harassment. Womens Stud Int Forum. 2016; 58(6): 9–17. doi: 10.1016/j.wsif.2016.04.001

[99] Cupach WR, Spitzberg BH. Obsessive relational intrusion and stalking. In Spitzberg BH, Cupach WR, editors. The dark side of close relationships. New Jersey: Erlbaum; 1998. pp. 233–263.

[100] Nguyen LK, Spitzberg BH, Lee CM. Coping with obsessive relational intrusion and stalking: the role of social support and coping strategies. Violence vict. 2012; 27(3): 414–433. doi: 10.1891/0886-6708.27.3.414 22852440

[101] Sani A, Nunes LM. [Security/insecurity diagnosis. An exploratory study in an urban community]. Anu Psicol Jurid. 2016; 26(1): 102–106. doi: 10.1016/j.apj.2015.07.001 Spanish.

[102] Skubak M, Eck JE. Getting a handle on crime: A further extension of routine activities theory. Secur J. 2011; 24: 179–193. doi: 10.1057/sj.2010.2

[103] Fox C, Asquith NL. Measuring the tangible fear of heterosexist violence. J Interpers Violence; 2018; 33(6): 980–1007. doi: 10.1177/0886260515614279 26611615

