# Supplementary material for: From harassment to disappearance: Young women’s feelings of insecurity in public spaces
Source: PLoS One. 2022 Sep 7;17(9):e0272933. doi: 10.1371/journal.pone.0272933 (PMC9451059; doi:10.1371/journal.pone.0272933)
Supplement: S1 Appendix — (DOCX) [file pone.0272933.s001.docx]

**Interview guide**

In recent years, insecurity has been frequently mentioned both in the media and in everyday conversations. We are interested in understanding your point of view on the issue of insecurity, and how it is presented in the city.

1. What is "insecurity" for you?

2. How unsafe is living in Mexicali?

3. What situations that occur in your neighborhood concern you? (Crimes, violent acts, other situations)

4. What situations that occur near your school concern you? (Crimes, violent acts, other situations)

5. What situations that occur in other zones of the city concern you? (Crimes, violent acts, other situations)

6. Which of these situations have you experienced?

7. Which of these situations have your acquaintances experienced?

8. What were the people who committed these acts like (crimes, violence, other situations)?

9. How do you feel about these situations (crimes, violent acts, other)?

10. In what places do you feel at risk? In what situations do you feel at risk? What do you think might happen to you?

11. What has changed in your life due to these situations (crime, violence, other)? How do you think it has affected you? What have you stopped doing?

12. What do you do to protect yourselves from these situations (crime, violence, other)?

13. What do you think should be done so that these situations do not occur? Who should do it?
